# Superabsorbent polymers seed coatings modulate transcriptomic and physiological responses to drought in rapeseed

**DOI:** 10.3389/fpls.2026.1711479

**Published:** 2026-02-11

**Authors:** Akram Abdolmaleki, Hendrik Bertram, Peter Dapprich, Elena Meininghaus, Marc Boelhauve, Michaela Schmitz, Armin O. Schmitt, Mehmet Gültas

**Affiliations:** 1 Faculty of Agriculture, South Westphalia University of Applied Sciences, Soest, Germany; 2 Breeding Informatics Group, Department of Animal Sciences, Georg-August University, Göttingen, Germany; 3 Center for Integrated Breeding Research (CiBreed), Georg-August University, Göttingen, Germany

**Keywords:** *Brassica napus*, drought stress, germination, SAP, seed coating, stress-responsive genes, transcriptomics

## Abstract

Drought stress is a major constraint on rapeseed (*Brassica napus* L.) production, particularly during germination and early seedling development, and its impact is intensifying with climate change. Superabsorbent polymers (SAPs) have emerged as a promising strategy to mitigate water limitation by enhancing moisture availability. This study conducted a comparative analysis of three SAP types, two fossil-based (MERCK, SWT) and one natural-based (ABG), applied via seed coating to evaluate their effects on germination, sodium uptake, total phenol content mitigation, and transcriptomic profiles under drought stress. While all SAPs increased seedling sodium content, the MERCK treatment produced the highest rate of normal germination, the lowest Na^+^ accumulation, and reduced oxidative stress, closely resembling the well-watered control (CN). Transcriptome sequencing revealed distinct expression profiles across treatments. MERCK seedlings showed expression of key stress-responsive genes (PER45, ABI1, STM) most similar to CN. In contrast, ABG seedlings exhibited significant downregulation of important genes (especially transcription factor (TF) genes) such as WRKY33, MYB77, CIPK17, and STZ, consistent with their poor performance. Functional enrichment analysis indicated the induction of phenylpropanoid biosynthesis, antioxidant activity, and hormonal signaling pathways, with MERCK and ABG showing contrasting signatures. These findings demonstrate that SAP composition influences drought adaptation in rapeseed by modulating molecular stress-response pathways. The integration of physiological and transcriptomic analyses not only identifies effective SAP formulations for seed coating but also provides candidate genes to support breeding programs aimed at developing stress-resilient cultivars.

## 1 Introduction

Rapeseed (*Brassica napus* L.) is the second-most cultivated oilseed crop globally, contributing approximately 15% of the total vegetable oil production, which is used for both human consumption and biofuel applications (Batool et al., 2022b). Its cultivation often has extended into arid and semi-arid regions, making it particularly vulnerable to drought stress, a major limiting factor for agricultural productivity worldwide (Kordrostami and Mafakheri, 2020; Dousti et al., 2025; Moradi Aghdam et al., 2019). Although rapeseed possesses some adaptive mechanisms, such as reactive oxygen species (ROS) scavenging and osmotic adjustment, it remains susceptible to water deficit, which can severely impair growth, yield, and seed quality (Channaoui et al., 2019; Li et al., 2025a). Drought disrupts key physiological processes at all developmental stages, from poor germination and inhibited seedling growth to reduced photosynthetic efficiency and nutrient uptake, largely due to osmotic stress and oxidative damage (Khan et al., 2019; Ai et al., 2024). The pronounced vulnerability of rapeseed’s early life stages to drought underscores the urgent need for innovative strategies to enhance crop resilience.

Among these developmental stages, seed germination is a particularly critical and sensitive phase. This process follows a tightly regulated triphasic pattern of water uptake: an initial rapid imbibition (phase I), a plateau phase for metabolic activation (phase II), and a final phase of water uptake leading to radicle emergence (phase III) (Mohamed et al., 2022). Drought stress directly disrupts this sequence by limiting water availability during imbibition, thereby impairing essential metabolic pathways and hindering the transition to autotrophic growth. The consequences of failed or delayed germination are severe, leading to poor stand establishment and cascading adverse effects on subsequent plant development, ultimately compromising yield and seed quality (Batool et al., 2022a). Therefore, interventions that secure water availability during this critical germination window are promising for enhancing rapeseed performance under drought conditions.

To improve crop establishment under water stress conditions, seed coating technology has emerged as a targeted and efficient solution. By creating a beneficial microenvironment around the seed, these coatings can enhance germination, emergence, and seedling survival (Koutzoukis et al., 2023). A key innovation for drought mitigation is the incorporation of moisture-absorbing materials such as superabsorbent polymers (SAPs). These hydrophilic, three-dimensional network polymers absorb and retain high amounts of water, forming a hydrogel that provides a sustained water supply directly to the seed and emerging radicle (Hoseini et al., 2022; Gorim and Asch, 2017).

While SAPs are often applied as soil amendments, this has notable drawbacks, including competition with plant roots for water (Sepehri et al., 2023), the release of sodium ions, and disruptive cation interactions in the soil (Zhang et al., 2014b; Liu et al., 2014). Furthermore, our recent work demonstrated that soil-applied SAPs had a moderate but highly variable effect on key plant traits, with efficacy differing significantly between species and even between cultivars (Abdolmaleki et al., 2025). This variability complicates the formulation of reliable recommendations for large-scale agricultural use, indicating that a more targeted delivery method may be preferable. Seed coating provides such a targeted solution. Although it represents only about 10% of SAP applications (Zheng et al., 2023), this method precisely places the polymer’s water storage capacity around the seed, where it is most needed during germination and seedling establishment. This localized application is not only more resource-efficient, requiring lower application rates than soil treatments (Hoseini et al., 2022), but has also been shown to enhance germination and early growth in various crops, including *Caragana korshinskii*, maize, red clover, and barley (Su et al., 2017; Pathak and Ambrose, 2019; Amirkhani et al., 2023; Gubišová et al., 2024). However, despite these promising findings, a comprehensive understanding of how different SAP coatings function for rapeseed remains elusive, as the underlying physiological and molecular mechanisms are poorly understood.

This knowledge gap highlights the need to bridge the gap between short-term technological advancements and long-term genetic enhancements. SAP seed coatings not only protect seedlings but also provide a model system for dissecting genetic pathways that mediate drought survival. By moderating extreme stress, coatings allow the identification of genes expressed under survivable conditions. Advances, such as the improved rapeseed reference genome (Lu et al., 2019) and high-throughput mRNA sequencing (Wang et al., 2021a; Liu et al., 2015; Guo et al., 2017), now enable the precise detection of differentially expressed genes (DEGs) that regulate drought tolerance. Previous transcriptomic studies have explored plant responses to nitrogen utilization (Chen et al., 2024), herbicides (Shah et al., 2022; Baek et al., 2019; Shi et al., 2020; Kaur et al., 2024; Chandra and Leon, 2022), and pesticides (Parakhia et al., 2019; Guo et al., 2020), identifying stress-responsive genes useful for crop improvement. Building on this approach, we investigated the effects of three SAP types—two fossil-based (MERCK, SWT) and one natural-based (ABG)—on rapeseed seedlings under drought stress. To address this, we employed an integrated, multi-layered strategy. We quantified germination and seedling establishment under well-watered and drought conditions to assess the efficacy of SAPs. We then performed transcriptome analysis to identify the genetic basis of SAP-mediated drought resilience. To further understand the underlying molecular mechanisms, we conducted Gene Ontology (GO) and Kyoto Encyclopedia of Genes and Genomes (KEGG) enrichment analyses, identifying the key biological processes and metabolic pathways associated with drought-responsive genes. These findings provide potential candidate genes for the development of molecular markers to be used in future breeding schemes.

Our physiological assessment showed that among the SAP treatments, the MERCK formulation performed best, producing the highest proportion of normal seedlings, the lowest rates of abnormal or ungerminated seeds, and reduced total phenolic content (TPC) and sodium (Na^+^) levels. Transcriptomic profiling linked these outcomes, indicating that MERCK not only enhances germination rate under drought but also activates stress-resistance genes, underscoring its dual role as a practical agronomic solution and a platform for genetic discovery.

## 2 Materials and methods

### 2.1 SAP treatments and application

Three distinct SAPs were evaluated for their efficacy as seed coatings. The application rate for each SAP was determined based on a combination of factors, including manufacturer recommendations, the observed water absorption capacity (**Table 1**), the thousand-seed weight (which for this variety is 3.5 g, reflecting the average mass of 1,000 seeds), and results from preliminary optimization tests. The selected polymers and their corresponding final application rates were:

**Table 1:** Water absorption capacity and comparative application rates of various SAPs.

SAP	Water absorption capacity		Usage amount per hectare (4 kg seeds)	
	Theoretical capacity (mL/g)	Observed capacity (mL/g)	Seed coating (kg)	Soil application (kg)
MERCK	500	470	0.114	50-70
SWT	600	250	0.228	65
ABG	10-40	10	4.516	400

The table presents the theoretical capacity (manufacturer’s description) and observed water absorption capacities (mL/g) of SAPs, along with the corresponding SAP application rates required for 1 hectare of land (soil application), based on the application of approximately 4 kg of seeds per hectare. Both theoretical and observed absorption capacities were determined using distilled water.

- MERCK: A fossil-based polymer (MERCK, Germany, Product No: MKCR9032) applied at 28.5 g per kg of seed.
- SWT: A fossil-based polymer (Isonem Soil Water Trap; Isonem, Turkey, Lot No: 55874) applied at 57.0 g per kg of seed.
- ABG: A natural-based polymer (AgroBioGel; AgroBiogel GmbH, Austria) applied at 1129 g per kg of seed.

### 2.2 Plant material and experimental design

The rapeseed cultivar (*Brassica napus* L. *subsp. napus* var. *napus f. biennis Thell.*, accession number CR 3261, Germany) was provided by the Leibniz Institute of Plant Genetics and Crop Plant Research (IPK). To assess the effect of SAPs on germination and gene expression under drought, the experiment was designed with five treatments: three SAP treatments (seeds coated with Merck, SWT, and ABG) and two uncoated control groups. The controls were a well-watered control (CN) grown under optimal moisture, and a drought stress control (CS) subjected to the same stress level as the SAP treatments. For the germination assay, ten coated seeds were germinated per petri dish on a double layer of filter paper (Filter Discs (Qual), grade 3 hw, diameter 90 mm, 65 g/m^2^, Göttingen, Germany). Drought stress was induced by applying a limited water volume of 2 mL to the CS and all SAP treatment groups, while the CN group received 5 mL (Rajendra Prasad, 2023). Each treatment had ten replicates and was incubated in a climate-controlled chamber at 25 *±* 1°C (BINDER Wachstumsschrank KBW 720, Tuttlingen, Germany) under a 16/8 h light/dark cycle for 7 days.

### 2.3 Germination traits

The effects of the SAP treatments on seed germination were assessed 7 days after sowing by categorizing seedlings into one of three classes according to criteria adapted from the International Seed Testing Association (ISTA) rules (Rajendra Prasad, 2023). Seedlings with a radicle length exceeding 2 mm, a straight primary root, and no visible necrosis on cotyledons or root tissues were classified as normal. Seedlings exhibiting developmental defects, such as a looped primary root, missing essential structures, or significant tissue necrosis, were classified as abnormal. Seeds with no visible signs of germination or with a radicle protrusion of less than 2 mm were classified as non-germinated. The number of seedlings in each category was quantified for every replicate. A multinomial-logistic model was fitted by the *multinom* function of the *nnet* (Venables and Ripley, 2002) package in R (R Core Team, 2021) using the treatments as the explanatory variable and the outcomes as the response (normal, abnormal, and non-germinated). The global treatment effect was tested with a likelihood-ratio test (LRT) using *Anova* of the *car* (Fox and Weisberg, 2019) package. Marginal probabilities were estimated by *emmeans* (Lenth, 2025), where pairwise treatment contrasts within each outcome were adjusted for multiple testing using Tukey’s correction and considered significant at *α* = 0.05.

Three separate tissue samples were collected from the normal seedlings, immediately flash-frozen in liquid nitrogen, and stored at -80°C. These samples were designated for the determination of TPC, and Na^+^ content, and for mRNA extraction.

### 2.4 Total phenol content determination

To prepare the sample, 200 mg of normal seedlings were flash-frozen in liquid nitrogen for 3 minutes and then pulverized for 10 minutes at 25,000 rpm in a mixer mill (Retsch Mixer Mill MM200, Haan, Germany). The resulting powder was subjected to a three-step extraction process. For each step, 1 mL of 95% methanol (CH_3_OH) was added to the powder, and the mixture was centrifuged at 5,000 rpm and 4°C for 5 min (Rotina 380R, Hettich, Kirchlengern, Germany). The supernatants from all three extractions were pooled to obtain the final methanolic plant extract. A reaction mixture was prepared for the colorimetric assay by combining 0.5 mL of deionized water, 0.5 mL of Folin–Ciocalteu reagent, and 0.5 mL of the methanolic extract. After mixing for 30 seconds, 5 mL of 1% sodium hydroxide (NaOH) solution was added, and the mixture was vortexed thoroughly. The solution was incubated for precisely 30 minutes at room temperature (20–22°C), and absorbance was measured at 760 nm using a UV-Vis spectrophotometer (UV-1800, Shimadzu, Duisburg, Germany) (Anuar, 2014). Finally, TPC content was quantified using a standard calibration curve prepared with gallic acid (see **Supplementary Section S1.1**).

### 2.5 Sodium content determination

Sodium content (Na^+^) was determined from normal seedlings. Sample extraction was performed following the guidelines provided by the manufacturer of the ion-selective electrode meter (LAQUAtwin Na^+^-11, Horiba, Magdeburg, Germany). Briefly, a 200 mg sample of fresh tissue was flash-frozen in liquid nitrogen and homogenized into a fine powder using the mixer mill for 15 minutes at 25,000 rpm. To the resulting powder, 0.5 mL of deionized water was added, and the mixture was vortexed thoroughly for 1 minute to ensure solubilization of sodium ions. The suspension was then centrifuged for 15 minutes at 1,000 rpm at room temperature (Rotina 380R, Hettich, Kirchlengern, Germany). The resulting supernatant was carefully collected, and its sodium concentration was measured directly using the pre-calibrated ion-selective electrode meter. All sample concentrations were recorded in parts per million (ppm).

Statistical analyses for both TPC and Na^+^ content were performed using R (R Core Team, 2021). Normality was assessed with the Shapiro-Wilk test (Shapiro and Wilk, 1965). Differences among treatment groups were then evaluated using a one-way analysis of variance (ANOVA), followed by Tukey’s Honestly Significant Difference (HSD) test for *post-hoc* pairwise comparisons. Group differences were considered to be significantly different across all analyses at *α* = 0.05.

### 2.6 mRNA extraction, library preparation, and data analysis workflow

Total RNA was extracted from normal seedlings using the TIANGEN RNAprep Pure Plant Kit (Cat. no. 4992237). Briefly, plant samples were ground to a fine powder in liquid nitrogen. For each 100 mg of tissue, 450 *µ*L of Buffer RL containing 1% *β*-mercaptoethanol was added. The mixture was vortexed vigorously and optionally incubated at 56 °C for 1–3 minutes. The lysate was transferred to an RNase-free filter column and centrifuged at 12,000 rpm for 2–5 minutes. The supernatant was collected for downstream processing. To ensure high RNA quality, DNase I treatment was carried out by adding 80 *µ*L of DNase I working solution (10 *µ*L of DNase I stock and 70 *µ*L of Buffer RDD) and incubating at room temperature for 15 minutes. The RNA was then purified through sequential washes with Buffer RW1 and Buffer RW, followed by elution with 30–100 *µ*L of RNase-free water. Total RNA was extracted from normal seedlings using the TIANGEN RNAprep Pure Plant Kit (Cat. no. 4992237, Beijing, China). Briefly, plant samples were ground to a fine powder in liquid nitrogen. For each 100 mg of tissue, 450 *µ*L of buffer RL containing 1% *β*-mercaptoethanol was added. The mixture was vortexed vigorously and optionally incubated at 56°C for 1–3 minutes. The lysate was transferred to an RNase-free filter column and centrifuged at 12,000 rpm for 2–5 minutes. The supernatant was collected for downstream processing. To ensure high RNA quality, DNase I treatment was carried out by adding 80 *µ*L of DNase I working solution (10 *µ*L of DNase I stock and 70 *µ*L of buffer RDD) and incubating at room temperature for 15 minutes. The RNA was then purified through sequential washes with buffer RW1 and buffer RW, followed by elution with 30–100 *µ*L of RNase-free water. RNA integrity was evaluated using the Agilent Bioanalyzer 2100 system (Agilent Technologies, Santa Clara, USA). mRNA was purified from total RNA using poly-T oligo-attached magnetic beads. After mRNA isolation and fragmentation, first-strand cDNA synthesis was performed using random hexamer primers. Second-strand synthesis was carried out using dUTP instead of dTTP to preserve strand specificity. Directional libraries were then constructed by performing end repair, A-tailing, adapter ligation, size selection, PCR amplification, and purification. The resulting libraries were quantified using a Qubit fluorometer and real-time PCR, and size distribution was assessed via the Bioanalyzer. After quality control, libraries were pooled based on effective concentration and target data yield, then subjected to Illumina sequencing. Sequencing was carried out using the sequencing-by-synthesis (SBS) method, in which fluorescently labeled dNTPs, DNA polymerase, and adapter primers are added to the flow cell for amplification and sequencing. As each cluster extends its complementary strand, a fluorescent signal corresponding to the incorporated dNTP is emitted and recorded by the sequencer. These signals are then processed into sequencing reads using built-in software to decode the sequence of the target fragment. The mRNA extraction and library preparation were performed by Novogene (Munich, Germany).

Initially, the raw sequence reads underwent quality control using fastp version 0.24.1 (Chen et al., 2018). Adapter sequences were automatically identified and removed via the–detect_adapter_for_pe parameter, and poly-G/poly-A tails were trimmed using –trim_poly_g and –trim_poly_x, respectively. Furthermore, reads were trimmed from the 3’-tails to the front using the options –cut_tail and –cut_mean_quality 20 to achieve window mean qualities above 20. Afterwards, reads shorter than 50 bases were discarded (–length_required 50). Finally, bases of reads were classified as unqualified if their phred-score was below 20 (–qualified_quality_phred 20), and reads were discarded if the number of unqualified bases exceeded 30% (–unqualified_percent_limit 30). Base and read quality statistics were generated using fastp (Chen et al., 2018) and FastQC version 0.12.1 (Andrews et al., 2010).

Reference genome, gene and protein annotations for the *Brassica napus* cv. Darmor-bzh reference genome *bnapus_darmor_bzh_v10* were downloaded from NCBI (Sayers et al., 2024) from accession number *PRJEB40412* (Rousseau-Gueutin et al., 2020) provided by Genoscope (Génoscope, 2024). Filtered reads were aligned to *bnapus_darmor_bzh_v10* using STAR version 2.7.11b (Dobin et al., 2012) with default parameters. Alignment statistics were generated using the bamqc and rnaseq commands of Qualimap version 2.3 (Okonechnikov et al., 2015). Consequently, all statistics were gathered using MultiQC version 1.28 (Ewels et al., 2016) for evaluation.

Sorting of alignment files was done using Sambamba version 1.0.1 (Tarasov et al., 2015) and gene-level quantification of alignment files was determined using featureCounts version 2.0.8 (Liao et al., 2013). Gene counts were extracted, and genes were filtered out if they were not abundant, e.g. if they did not have a raw count of at least 10 in at least 70% of the samples. Differential expression analysis using DESeq2 version 1.46.0 (Huber and Love, 2017) in R version 4.4.3 (R Core Team, 2021) was conducted with CS as the reference group, applying false discovery rate (FDR) adjustment for p-values (Benjamini and Hochberg, 1995) (padj) and log_2_ fold change (LFC) shrinkage based on apeglm (Zhu et al., 2018). Finally, for each comparison, we considered genes differentially expressed if padj *<* 0.05 and *|*LFC*| >* 1.

To obtain GO and KEGG annotations, eggNOG-mapper version 2.1.12 (Cantalapiedra et al., 2021) was run using DIAMOND (Buchfink et al., 2021) for protein alignment against the Brassicaceae family of the EggNOG version 5.0 (Huerta-Cepas et al., 2018) database. GO and KEGG Pathway descriptions were obtained from the KEGG database (Kanehisa, 2000) and the GO database (Aleksander et al., 2023), respectively. For each comparison, we performed functional enrichment of GO terms and KEGG Pathways based on the DEG sets by using the enricher function of clusterProfiler (version 4.14.6) (Guangchuang et al. (2017)). Terms and pathways were considered significantly enriched if padj *<* 0.05. *Arabidopsis thaliana* orthologs and gene names were retrieved from BnaOmics (Cui et al., 2023).

## 3 Results

### 3.1 SAPs effects on seed germination characteristics

**Figure 1:**
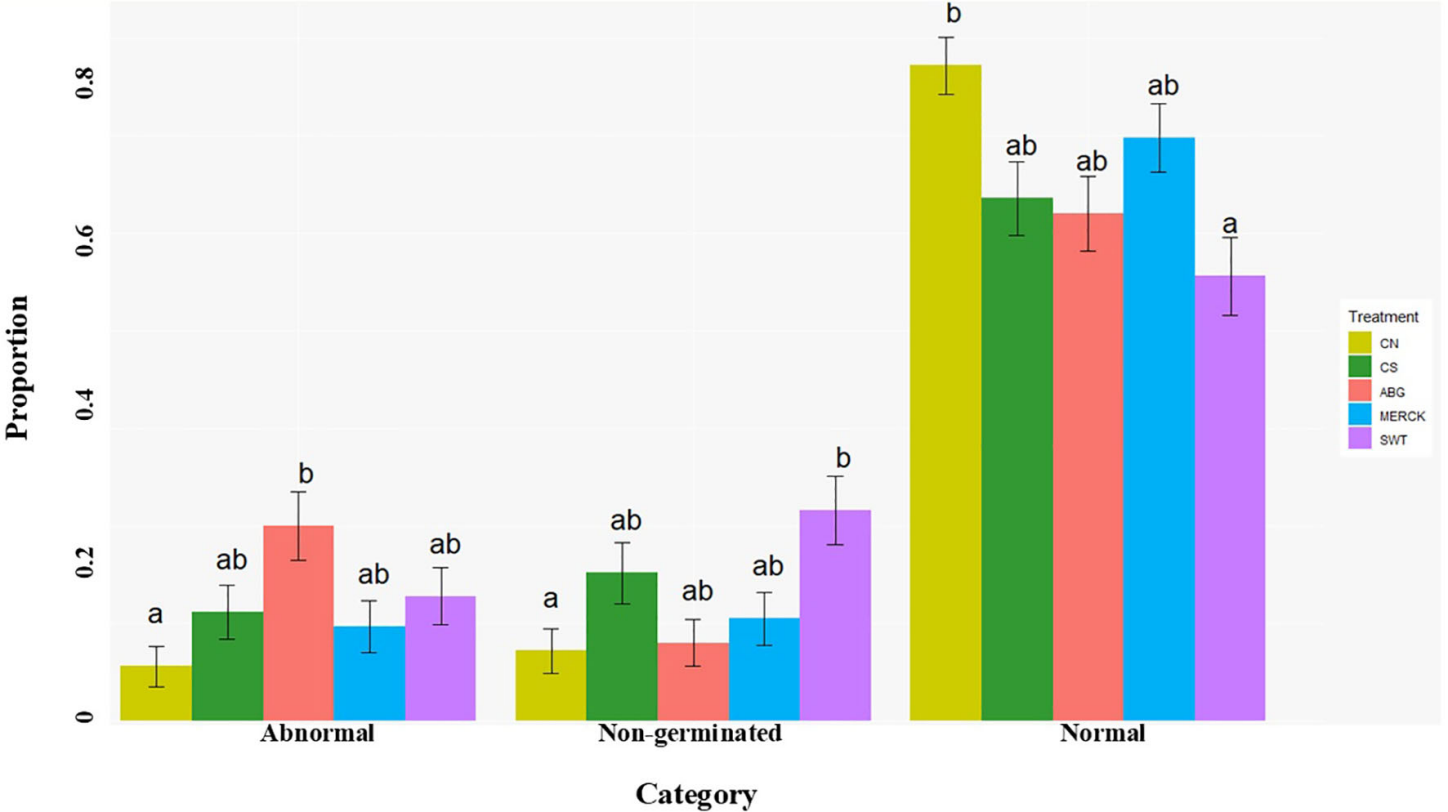
Effects of SAP treatments on seed germination traits. Bar plot showing the mean proportions (± SE) of abnormal, non-germinated, and normal seedlings across different SAP treatments (ABG, MERCK, SWT) and control groups (CN and CS). Statistical significance was assessed using a multinomial logistic model with a likelihood-ratio test. Distinct letters above the bars indicate significant differences among treatments within each germination outcome (*α* = 0.05).

The treatment effects on germination outcomes were found to be significant. *Post-hoc* pairwise comparisons were conducted to distinguish between treatment groups within each germination outcome (normal, abnormal, and non-germinated). The results, with significant differences (*α* = 0.05) indicated by compact letter displays, are shown in **Figure 1**.

The analysis highlighted distinct performance patterns among the treatments. As the baseline, the CN produced the highest proportion of normal seedlings and the lowest frequency of abnormal and non-germinated seeds. Among the SAP treatments, MERCK was the most effective, performing comparably to the CN group by maintaining a high proportion of normal seedlings and minimizing developmental abnormalities, thereby indicating successful mitigation of drought stress. The drought-stressed control (CS) exhibited an intermediate response. In contrast, the ABG treatment led to a higher incidence of abnormal seedlings, suggesting that it may impair developmental processes under stress. However, this effect was not always statistically significant compared to the controls. The SWT treatment demonstrated the poorest performance, resulting in the lowest proportion of normal seedlings and the highest rate of non-germinated seeds, indicating it failed to alleviate drought stress and may have even inhibited germination initiation. These findings identify MERCK as the most effective SAP treatment for promoting healthy and uniform seedling establishment in rapeseed under the tested drought conditions, with performance comparable to ABG but better than SWT.

### 3.2 TPC and Na^+^ content of seedlings

**Figure 2:**
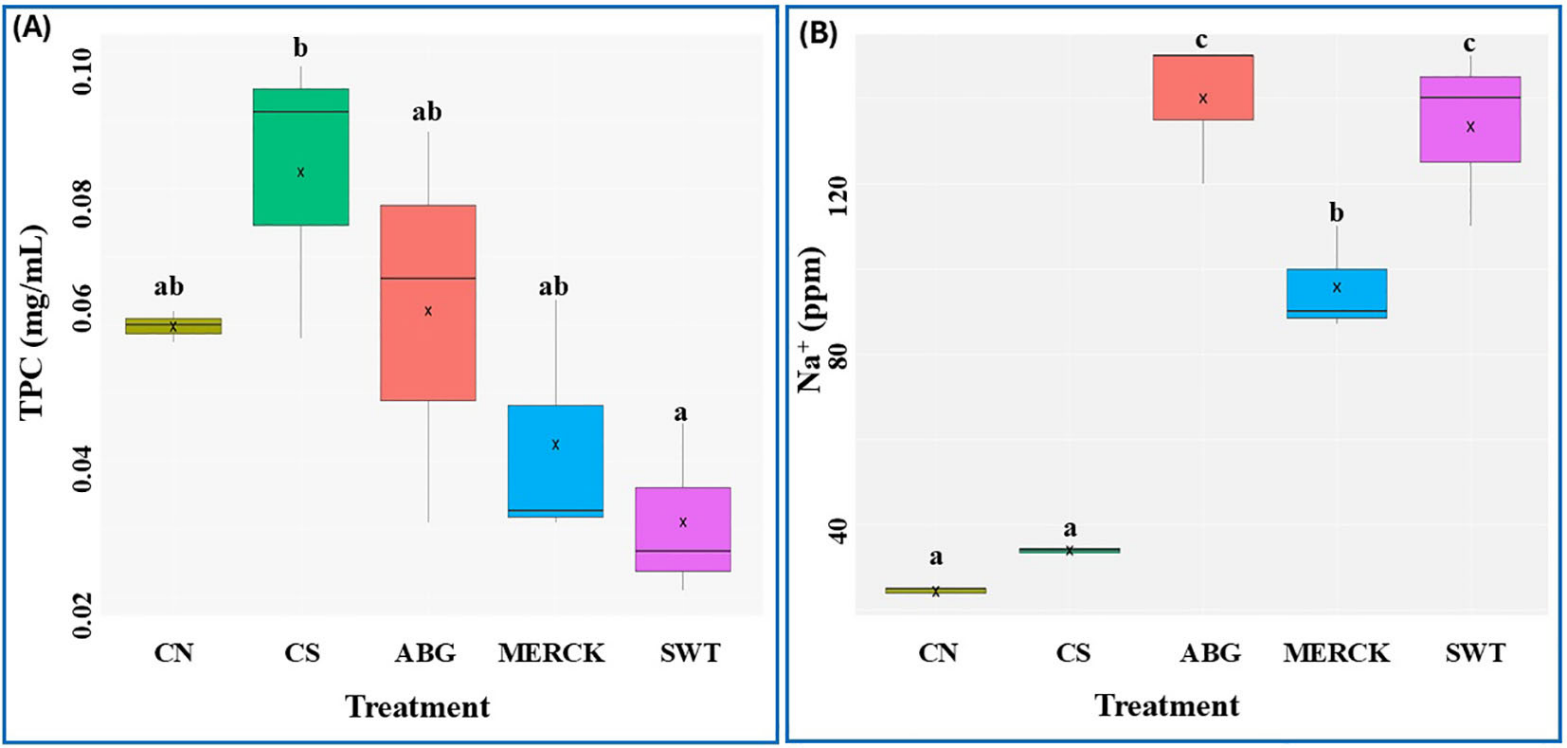
Total phenol content and sodium content analysis. The effects of treatments on **(A)** TPC and **(B)** Na^+^ concentration are presented. A one-way ANOVA was performed to assess overall treatment effects, followed by a Tukey’s *post hoc* test for pairwise comparisons. Distinct letters above the bars indicate statistically significant differences between treatments within each measured trait (*α* = 0.05). The ‘X’ symbol represents the mean value for each treatment.

The biochemical impact of the SAP treatments was first evaluated by measuring TPC, a key indicator of oxidative stress. A one-way ANOVA confirmed that the treatments had a significant effect on TPC levels in rapeseed seedlings (**Figure 2A**). Both fossil-based SAPs, MERCK and SWT, resulted in lower TPC accumulation compared to the CS and the natural-based ABG treatment. Notably, the mitigating effect was most pronounced in the SWT treatment, which maintained the lowest TPC of all drought-exposed groups. “Plants maintain a baseline level of TPC even under normal conditions (Singh et al., 2016), and consistent with this, the CN group also exhibited baseline TPC production in seedlings under optimal conditions. Among the SAP treatments, the natural-based ABG produced a lower TPC compared to CS but did not outperform CN. By contrast, the fossil-based polymers showed more favorable effects: MERCK induced lower TPC than both controls, while SWT reduced TPC to an even greater extent. The reduction of TPC in seedlings treated with these fossil-based SAPs, despite drought stress, suggests that such polymers may partially alleviate oxidative stress and thereby modulate the phenolic response.

To evaluate the ionic stress potentially introduced by the sodium-based polymers, seedling Na^+^ accumulation was quantified. A one-way ANOVA confirmed a highly significant treatment effect on seedling Na^+^ content (**Figure 2B**). As expected, both CN and CS control groups maintained low Na^+^ levels. In contrast, all three SAP treatments led to a significant increase in Na^+^ accumulation, confirming that the polymers introduce a sodium load to the seedlings. Among the SAP treatment groups, seedlings coated with MERCK accumulated significantly less Na^+^ (approximately 35%) compared to those treated with ABG or SWT, indicating a lower level of sodium transfer from this specific polymer.

Collectively, the biochemical data reveal a fundamental physiological constraint associated with the application of SAP. While the SAPs effectively mitigated drought-induced oxidative stress (lower TPC), they simultaneously introduced a significant ionic stress load (higher Na^+^). Notably, the MERCK treatment provided the most favorable balance, demonstrating both a strong reduction in oxidative stress markers and a significantly lower level of sodium accumulation compared to the other SAPs. This evidence suggests that MERCK is the most promising candidate for mitigating drought effects when applied as a seed coating, offering a short-term solution with reduced risk of secondary ionic stress.

### 3.3 Transcriptome analysis

A total of 10 libraries (two biological replicates for each of the five treatment groups) were sequenced, yielding between 122 and 194 million raw reads per sample (**Supplementary Table S1**). Following the removal of adapters and stringent quality filtering, over 98.0% high-quality reads were retained. Subsequent mapping of these clean reads to the *Brassica napus* reference genome resulted in high alignment rates, with 96.6% to 98% of reads successfully mapped for each sample.

**Figure 3:**
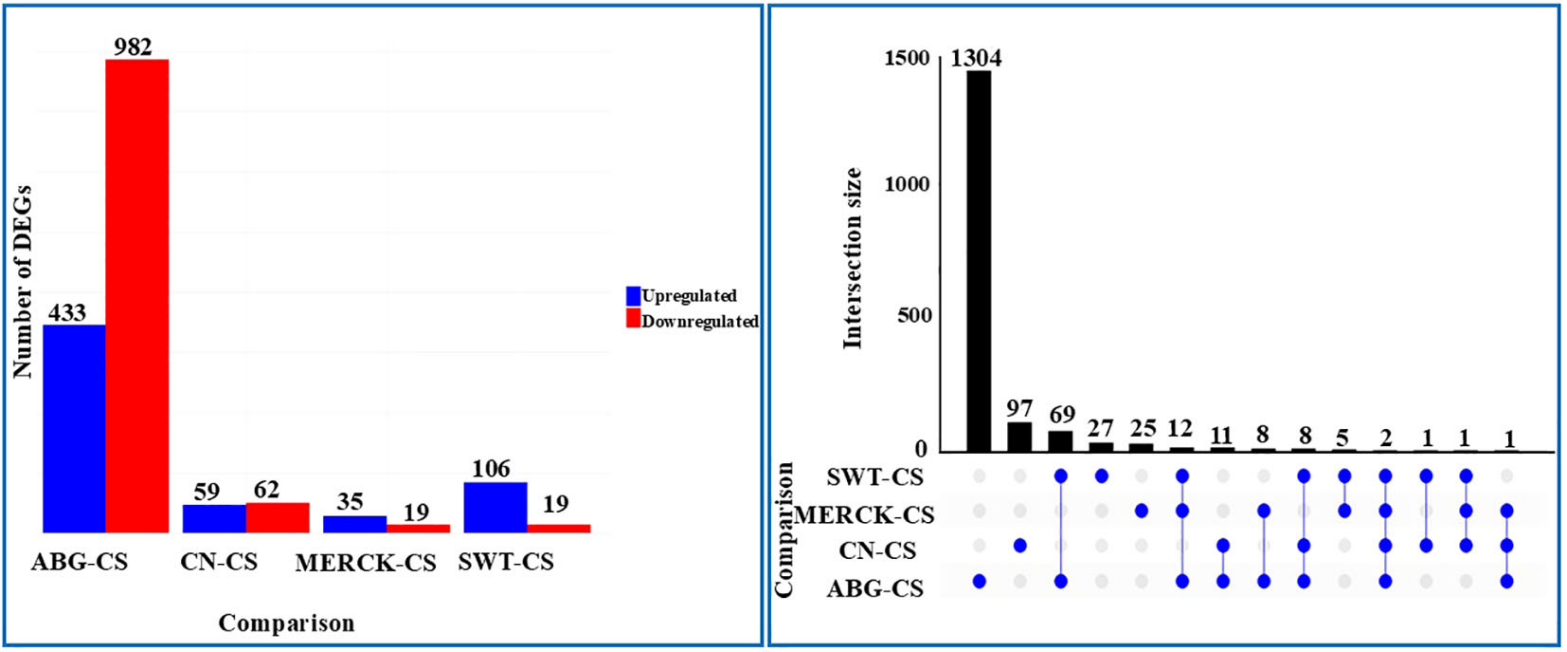
Transcriptomic variation across treatment groups relative to CS. **(A)** Number of DEGs identified in each pairwise comparison against CS as reference group, categorized by direction of regulation. **(B)** UpSet plot illustrating the intersection of DEGs among comparisons, depicting treatment-specific and shared transcriptional responses to SAP application.

Differential gene expression analysis revealed that each treatment elicited a unique transcriptomic signature in response to drought stress (**Figure 3A**). The most pronounced response was observed in the ABG treatment, which, when compared to the drought-stressed control (ABG vs. CS), resulted in 1,415 DEGs (433 upregulated, 982 downregulated). The comparison between the CN and stressed controls (CN vs. CS) identified 121 DEGs, establishing a baseline drought response. Notably, the MERCK treatment induced the smallest transcriptomic shift relative to CS (MERCK vs. CS), with only 54 DEGs identified (35 upregulated, 19 downregulated). This minimal response was substantially lower than that of the SWT treatment (SWT vs CS), which yielded 125 DEGs (106 upregulated, 19 downregulated). To dissect the specificity and overlap of the transcriptomic responses, we visualized the intersections of DEGs using an UpSet plot (**Figure 3B**). This analysis revealed that each treatment induced a largely unique genetic program. The ABG treatment dominated the response, with 1,304 DEGs unique to this polymer alone. The fossil-based SAPs triggered much smaller unique responses, with only 27 DEGs being to MERCK and 25 to SWT. In terms of shared effects among the SAPs, the largest overlap was between ABG and SWT (69 DEGs), while all three polymers commonly regulated a core set of only 12 DEGs. Although there is some overlap in DEGs, particularly for SWT and ABG, indicating shared molecular pathways, the overall response elicited by each SAP is distinct. When compared to the baseline drought response (CN vs. CS), we found that 24 of these DEGs were also modulated by at least one SAP treatment. Ultimately, only two genes were found to be common across all four comparisons, underscoring the distinct transcriptomic signatures of each SAP treatment relative to both one another and to the drought-stressed and well-watered conditions.

#### 3.3.1 GO enrichment analysis

**Figure 4:**
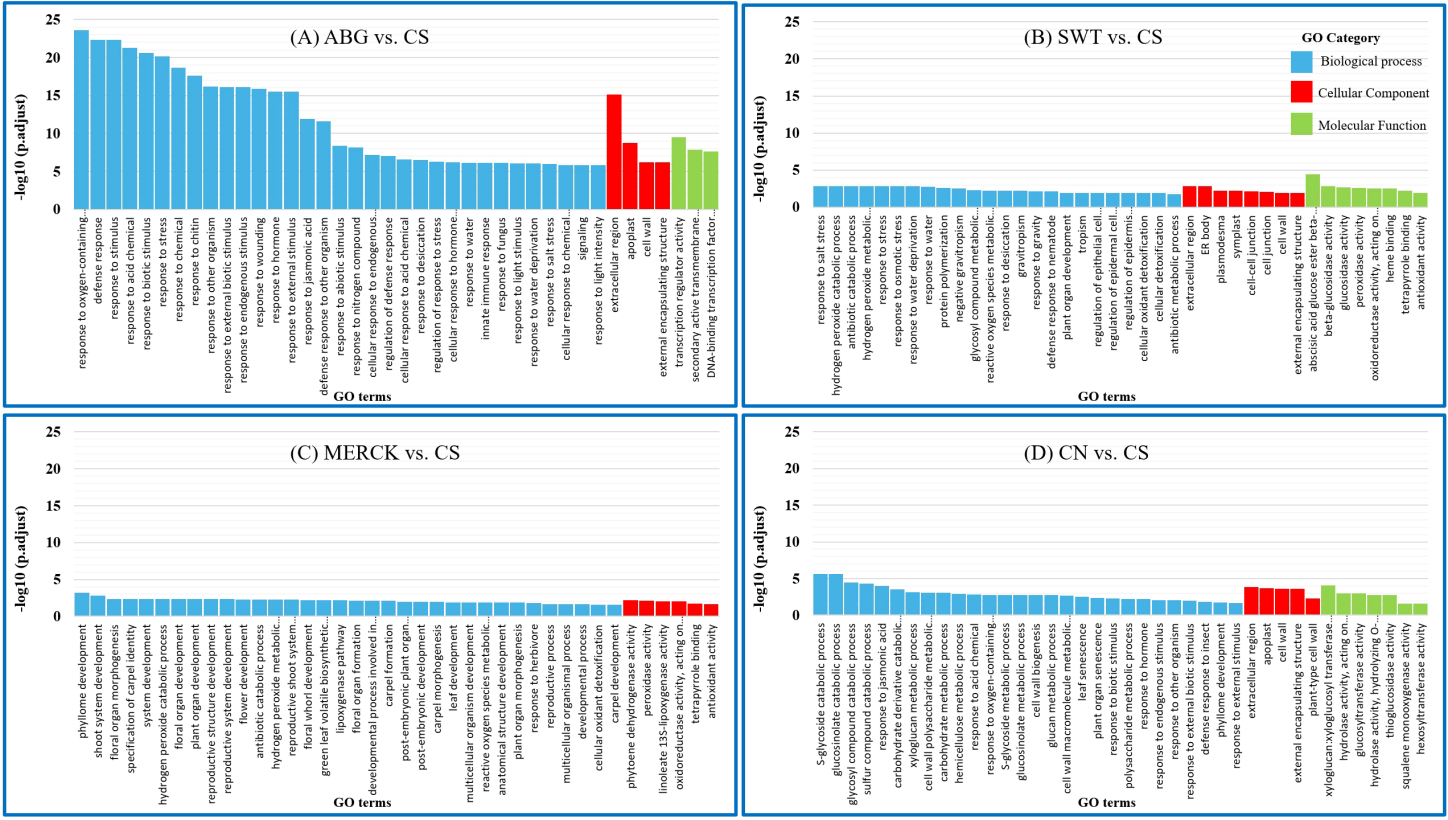
Top 40 significantly enriched GO terms associated with the DEGs in each treatment comparison group. Bars represent enriched GO terms classified into Biological Process, Cellular Component, and Molecular Function for the following contrasts: **(A)** ABG vs. CS, **(B)** SWT vs. CS, **(C)** MERCK vs. CS, and **(D)** CN vs. CS. GO terms are ranked by *−*log_10_(padj), highlighting key functional categories affected by SAP treatments under stress conditions.

GO enrichment analysis revealed both shared and unique biological processes activated by the different SAPs (**Figure 4**). A notable overlap was observed between the ABG vs. CS and SWT vs. CS treatments, which both induced pathways related to water deficit. These included key terms such as “response to stress” (GO:0006950), “response to water” (GO:0009415), and “response to osmotic stress” (GO:0006970). Particularly, the enrichment of “response to salt stress” (GO:0009651) in these treatments (**Figures 4A, B**) suggests that they may impose a secondary ionic stress on the seedlings. A common theme across all SAP treatments, including MERCK vs. CS, was the induction of oxidative stress responses. This was evidenced by the enrichment of terms like “reactive oxygen species metabolic process” (GO:0072593), “hydrogen peroxide catabolic process” (GO:0042744), and “cellular oxidant detoxification” (GO:0098869) (**Figure 4C**). This universal response indicates that seedlings activated antioxidant defenses, which are likely to mitigate the combined effect of water and ionic stress imposed by the polymers. Remarkably, no single GO term was commonly enriched across all treatment comparisons. However, specific overlaps pointed to distinct mechanisms. For instance, hormone-related pathways like “response to hormone” (GO:0009725) and “response to jasmonic acid” (GO:0009753) were shared between the ABG vs. CS and CN vs. CS comparisons. In contrast, terms associated with the cell wall and extracellular region [e.g., “cell wall” (GO:0005618), “external encapsulating structure” (GO:0030312)) were common to both the SWT vs. CS and CN vs. CS comparisons.

#### 3.3.2 KEGG enrichment analysis

KEGG pathway enrichment analysis of the DEGs revealed that the SAP treatments modulated several key metabolic and signaling networks (**Table 2**). The ABG treatment, which induced the largest transcriptomic shift, was uniquely associated with the enrichment of pathways related to environmental adaptation, including “Circadian rhythm - plant” (*bna04712*) and “Photosynthesis - antenna proteins” (*bna00196*). This suggests a broad reprogramming of energy acquisition and temporal regulation. Despite treatment-specific profiles, several core pathways were commonly enriched across SAP treatments. “Phenylpropanoid biosynthesis” (*bna00940*) and “Biosynthesis of secondary metabolites” (*bna01110*) were significantly enriched in all three SAP comparisons (ABG, MERCK, and SWT vs. CN), indicating that altered expression of defense-related secondary metabolism is a conserved response to SAP application. Furthermore, pathways involved in lipid signaling, “Linoleic acid metabolism” (*bna00591*) and “alpha-Linolenic acid metabolism” (*bna00592*), were commonly enriched in both the ABG and MERCK treatments. Wang et al. (Wang et al., 2021b) demonstrated that fatty acid metabolites can regulate stress tolerance in rapeseed seedlings, and the shared modulation observed in our study suggests that fatty acid–derived signaling molecules underlie a convergent mechanism of stress response between these two polymers.

**Table 2:** Enriched KEGG pathways across treatment comparisons.

Pathway ID	Description	P.adjust	RichFactor	Number of genes
ABG vs. CS				
bna04712	Circadian rhythm - plant	1.34E-11	0.140	24
bna04075	Plant hormone signal transduction	1.51E-11	0.052	70
bna01110	Biosynthesis of secondary metabolites	1.24E-05	0.031	127
bna00196	Photosynthesis - antenna proteins	3.67E-05	0.145	10
bna00591	Linoleic acid metabolism	4.62E-04	0.163	7
bna00630	Glyoxylate and dicarboxylate metabolism	4.62E-04	0.066	17
bna01100	Metabolic pathways	4.78E-04	0.026	186
bna00592	alpha-Linolenic acid metabolism	6.58E-04	0.082	12
bna00940	Phenylpropanoid biosynthesis	4.76E-03	0.049	19
bna04016	MAPK signaling pathway	4.76E-03	0.044	24
bna00460	Cyanoamino acid metabolism	1.68E-02	0.059	11
bna00500	Starch and sucrose metabolism	1.68E-02	0.043	19
bna00250	Alanine, aspartate and glutamate metabolism	2.05E-02	0.056	11
bna04626	Plant-pathogen interaction	4.38E-02	0.035	26
SWT vs. CS				
bna00940	Phenylpropanoid biosynthesis	1.50E-04	0.018	7
bna01110	Biosynthesis of secondary metabolites	4.23E-02	0.004	15
MERCK vs. CS				
bna00940	Phenylpropanoid biosynthesis	1.58E-03	0.010	4
bna00591	Linoleic acid metabolism	2.85E-03	0.047	2
bna01110	Biosynthesis of secondary metabolites	1.11E-02	0.002	8
bna00592	alpha-Linolenic acid metabolism	1.60E-02	0.014	2

KEGG pathways significantly enriched ( α=0.05) among DEGs in each treatment group relative to CS. P.adjust denotes false discovery rate (FDR)–corrected p-values based on the Benjamini–Hochberg method, and RichFactor represents the ratio of DEGs assigned to a pathway to the total number of genes annotated to that pathway. The number of genes refers to the number of DEGs associated with a given pathway.

Unlike the SAP treatment groups, the CN vs. CS comparison yielded no significantly enriched KEGG pathways. This indicates that the observed pathway-level modulations are a direct consequence of the SAP application rather than a general drought response, supporting the hypothesis that SAPs induce additional stress, particularly through sodium accumulation.

#### 3.3.3 Target genes analysis

**Figure 5:**
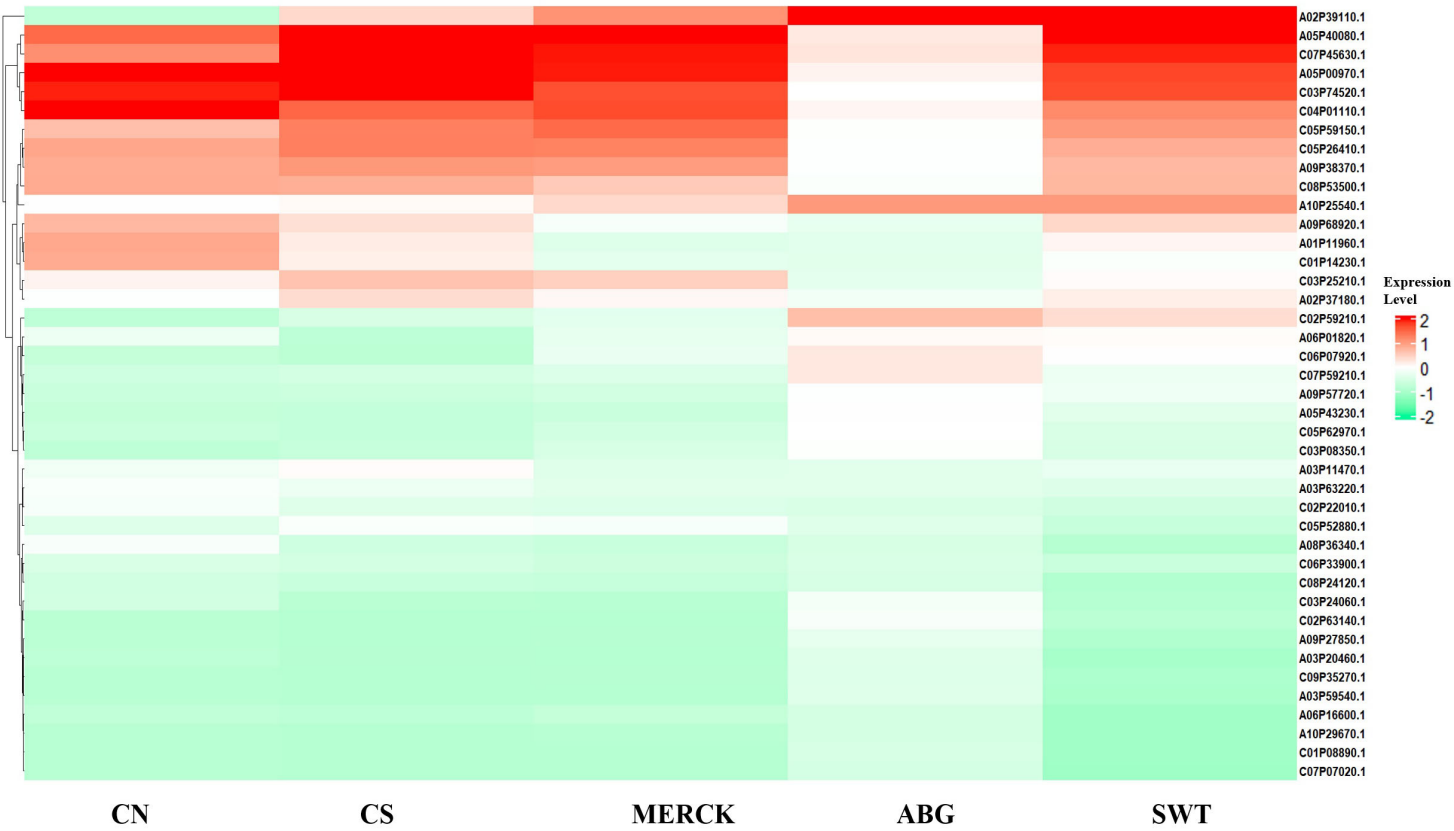
Heatmap of stress-responsive gene expression. Gene expression of selected stress-responsive genes annotated with GO terms related to salt stress, water deprivation, hydrogen peroxide catabolism, and antioxidant activity across SAP treatments and control groups.

The gene expression heatmap reveals distinct transcriptional signatures for each SAP treatment (**Figure 5**), focusing on genes from five key functional categories relevant to drought and ionic stress: “cellular response to toxic substance,” “response to water deprivation,” “response to salt stress,” “hydrogen peroxide catabolic process,” and “antioxidant activity.” A clear pattern emerged when comparing the treatments to CN. The natural-based ABG treatment exhibited a distinct expression pattern. In contrast, both fossil-based SAPs (MERCK and SWT) produced profiles more similar to CN, with MERCK showing the closest resemblance. Overall, these patterns suggest that ABG triggered a pronounced molecular stress response, whereas MERCK maintained a gene expression profile comparable to unstressed control seedlings.

A closer examination of regulation patterns revealed that MERCK treatment consistently mirrored the quiescent transcriptional state of the CN group (**Table 3**). SWT showed a broadly similar profile, but with exceptions where it induced strong gene upregulation (e.g., *A06P01820.1*). In contrast, ABG triggered numerous genes with significant positive (e.g., *A08P36340.1*) or negative log fold change (LFC) values, indicative of a pronounced stress response.

**Table 3:** Differential expression of stress-responsive genes under drought.

Gene ID	Arabidopsis ortologs	Gene name	Log2FoldChange			
			CN vs. CS	MERCK vs. CS	ABG vs. CS	SWT
C06P07920.1	AT1G52400	BGLU18	0.03	2.81^*^	3.84^*^	3.28^*^
A06P01820.1	AT1G52400	BGLU18	2.24	1.91	2.85^*^	2.68^*^
C03P25210.1	AT2G38470	WRKY33	-0.02	0.00	-1.97^*^	0.00
C03P74520.1	AT1G27730	STZ	-0.01	0.00	-1.78^*^	0.00
C05P52880.1	AT3G14440	NCED3	-0.03	0.00	-1.68^*^	0.00
C07P45630.1	AT3G50060	MYB77	-0.03	0.00	-1.13^*^	0.00
C08P53500.1	AT1G48260	CIPK17	-0.01	0.00	-1.18^*^	0.00
C06P33900.1	AT1G73680	ALPHA DOX2	0.00	0.00	-1.41^*^	0.00
A09P38370.1	AT1G27730	STZ	-0.02	0.00	-1.25^*^	0.00
A03P11470.1	AT5G59820	RHL41	-0.02	0.00	-1.70^*^	0.00
A01P11960.1	AT4G20260	PCAP1	0.01	0.00	-1.83^*^	0.00
C01P14230.1	AT4G20260	PCAP1	0.01	0.00	-1.75^*^	0.00
A03P63220.1	AT4G35000	APX3	0.00	0.00	-1.15^*^	0.00
A08P36340.1	AT1G05680	UGT74E2	0.04	0.00	-2.73^*^	0.00
C01P08890.1	AT4G30170	PER45	0.00	-5.96^*^	4.24^*^	2.71^*^
A06P16600.1	AT1G20620	CAT3	0.01	0.00	-2.66^*^	0.00
C02P22010.1	AT1G56650	PAP1	0.01	0.00	-1.64^*^	0.00
A05P43230.1	AT3G04120	GAPC1	0.00	0.00	1.86^*^	0.00
A05P40080.1	AT3G08730	PK1	-0.02	0.00	-1.23^*^	0.00
C07P59210.1	AT4G32410	CESA1	-0.02	0.00	1.31^*^	0.00
A10P25540.1	AT5G12250	TUB6	-0.05	0.00	1.00^*^	0.00
C08P50590.1	AT4G26080	ABI1	-0.03	1.99^*^	0.85	0.00

*Arabidopsis thaliana* orthologs and their gene names are listed for functional context where available. Negative log2 fold changes (LFC) denote downregulation (green) and positive LFC-values denote upregulation (red) relative to CS. Asterisks (*) indicate significant differences in LFC, based on padj *<* 0.05 and *|*LFC*| >* 1.

The expression patterns of stress response genes serve as valuable molecular indicators of a plant’s physiological response to environmental challenges. In this study, the analyzed gene set encompasses a diverse array of biological processes and molecular functions essential for plant adaptation to abiotic stresses, including drought, osmotic imbalance, oxidative stress, and hormonal disruption. These genes can be categorized into key functional groups, e.g., ROS detoxification (e.g., *APX3, CAT3, PER64, PER39*), hormone biosynthesis and signaling (e.g., *NCED3, UGT74E2, PAP1*), transcriptional regulation (e.g., *WRKY33, MYB77, STZ, RHL41*), metabolic adjustment (e.g., *GAPC1, PK1, ALPHA DOX2*), and cell wall biosynthesis and remodeling (e.g., *CESA1, TUB6, PRX69*) (**Table 2**). These pathways collectively enable plants to perceive and respond to various abiotic challenges, including salinity, drought, and oxidative damage.

This disordered expression profile indicates that, although the plants attempted to defend themselves, the response was likely weak or ineffective, leaving unresolved physiological stress from ionic, oxidative, and water-related sources. Ultimately, this differential regulation highlights the highly variable capacity of these SAPs to effectively modulate plant stress responses, with some treatments providing clear benefits. In contrast, others may compromise the plant’s overall defense strategy.

Analysis of common DEGs among all SAPs showed that most are stress-related (**Supplementary Table S2**). All were significantly upregulated in SAPs compared to CN, except *A04P33570.1*, which was consistently downregulated, and *C01P08890.1*, which was downregulated only in MERCK. This suggests that while SAPs share similarities, variation in the expression levels of these common genes may contribute to phenotypic differences and performance variation among SAPs. All DEG datasets and GO enrichment analysis results are provided in the supplementary files (**Supplementary Tables S3**, **S4**).

## 4 Discussion

Seed germination, early seedling development, and establishment are critical for successful crop production. These developmental phases are highly sensitive to abiotic stresses, which can compromise seedling vigor and survival (Liu et al., 2024). Among these stresses, drought is particularly detrimental, severely inhibiting plant growth and development, and ultimately resulting in significant yield losses (Fang et al., 2022).

### 4.1 The dual physiological impact of SAPs: sodium stress and the antioxidant response

High sodium concentrations are well-documented to impact seed viability and germination negatively. For example, studies have shown that increased Na^+^ levels are significantly correlated with decreased germination percentage and germination rate (Aloui et al., 2025; Zhang et al., 2014a). Our analysis of seedling sodium content confirmed this interaction, revealing that all SAP treatments significantly increased Na^+^ accumulation in the plant tissues (**Figure 2B**). This indicates that, in addition to their water-retaining function, these SAPs also act as a source of sodium stress for the seedlings. In agreement with other studies demonstrating the negative consequences of excess sodium (An et al., 2025; Gul et al., 2025), this release of sodium offers a mechanistic explanation for the adverse developmental outcomes we observed. The resulting ionic and osmotic stress is a well-known cause of germination inhibition and developmental abnormalities in seedlings. As such, we propose that the high rates of non-germination and abnormality observed in our experiments (**Figure 1**) are likely a consequence of this SAP-induced sodium stress. Notably, MERCK accumulated the least amount of sodium of all SAPs. This lower stress level likely led to better developmental outcomes, as these seedlings also exhibited a high rate of normal germination and a low rate of abnormalities. Hence, the observed resilience under the MERCK treatment can probably be attributed to the induction of a specific gene expression program that initiates molecular pathways to compensate for the imposed but manageable salinity stress.

Furthermore, the accumulation of phenolic compounds, quantified as TPC, is a well-established plant response to oxidative stress induced by environmental challenges such as drought (Saini et al., 2024). Consistent with this, studies have reported enhanced antioxidant production and elevated TPC in drought-stressed crops like rapeseed and maize (Ayyaz et al., 2022; Zhang et al., 2020). These secondary metabolites function as crucial non-enzymatic antioxidants, directly scavenging ROS to mitigate cellular damage and maintain redox balance (Tanveer et al., 2023). Consequently, TPC levels can serve as a reliable indicator of the physiological stress experienced by the plant; an increase in TPC reflects an alleviated stress state, while a decrease suggests effective stress mitigation. We observed that all SAPs reduced mean TPC relative to the CS control, but with high within-group variances. The fossil-based formulations were most effective, with SWT producing the most significant reduction compared to CS, followed by MERCK, while ABG showed only a modest decrease. Because TPC typically rises with ROS-driven phenylpropanoid induction, these declines indicate attenuated oxidative signaling under SAPs. Overall, the results suggest that SAPs, particularly those based on fossil chemistries, mitigate specific oxidative stresses and thereby protect plants.

### 4.2 Transcriptome analysis

Our transcriptome analysis revealed that different SAPs elicited distinct molecular responses, thereby establishing a genetic foundation for the observed phenotypic differences. We identified many stress-responsive genes, whose expression patterns suggest the necessary transcriptional reprogramming to endure drought and ionic stress. A primary distinction emerged between the fossil-based polymers (MERCK, SWT), which induced modest, directed changes in gene expression, and the natural-based polymer (ABG), which elicited a markedly stronger response, likely reflecting salt-induced dysregulation. To explore these differences, we will first discuss the unique adaptive strategy prompted by fossil-based polymers, followed by an analysis of the stress response activated by natural-based polymers.

#### 4.2.1 A. Fossil-based SAPs

A particularly striking finding was the divergence between the MERCK treatment and CN. Although MERCK-treated seedlings achieved a high rate of normal germination similar to the control, their transcriptomic profiles differed markedly from CN, with limited overlap in DEGs or enriched pathways (**Figures 4C, D**). This observation suggests that MERCK does not simply stabilize the environment to maintain homeostasis; rather, it induces a proactive and compensatory transcriptional program. This unique gene expression signature appears to mitigate the imposed stress, enabling germination to proceed.

Hormonal and developmental regulators exhibited treatment-specific control. The peroxidase ortholog *C01P08890.1* (*PER45, AT4G30170*) (**Table 3**), a multifunctional enzyme involved in H_2_O_2_ detoxification, cell wall fortification, and auxin catabolism (Passardi et al., 2005; Gómez-Sagasti et al., 2023; Vatansever et al., 2016; Roomi et al., 2018), was significantly downregulated in MERCK but induced in ABG and SWT. This inhibition likely reduced auxin degradation, conserving hormonal pools necessary for developmental progression. At the same time, meristem regulators *STM* (*C09P19820.1*) and *KNAT2* (*C02P27790.1*), central to shoot apical meristem maintenance and stress adaptation (Lopes et al., 2024; Zhang et al., 2025; Elhiti et al., 2012; Lechon et al., 2025; Lee et al., 2016; Song et al., 2021), were consistently upregulated. Their activation suggests reinforcement of meristem robustness, sustaining continuous organ formation even under stress. Evidence of ABA signaling modulation was also apparent. The *ABI1* ortholog (*C08P50590.1*), a negative regulator of ABA signaling (Leung et al., 1997), was significantly upregulated in MERCK. In rapeseed, *ABI1* is known as a hub gene modulating salinity and drought tolerance (Shamloo-Dashtpagerdi et al., 2022). The induction observed under MERCK suggests a strategy of defense restraint, in which ABA signaling was maintained in an active state but prevented from escalating into growth arrest. This balance likely enabled seedlings to cope with stress while sustaining germination.

Stress-modulating enzymes, particularly the *β-glucosidase* (*BGLU*) family, displayed clear divergence between treatments, highlighting their role in mediating stress outcomes. *BGLUs* are key modulators of the ABA stress response, releasing bioactive ABA from its inactive, stored form (ABA-glucose ester) (Han et al., 2019). The SWT treatment, which resulted in the most severe outcome with the highest rate of seedling abnormality, exhibited strong upregulation of the *BGLU* orthologs *C06P07920.1* and *A06P01820.1*. This suggests an intensified and likely uncontrolled mobilization of ABA, leading to a level of hormonal stress that impaired normal germination and development (Han et al., 2019). In addition, the MERCK treatment, which produced a high proportion of normal seedlings despite significant ionic stress, maintained lower upregulated expression of these *BGLU* genes. By avoiding the excessive, *BGLU*-mediated release of active ABA (Zhang et al., 2024; Karssemeijer et al., 2022; Mo et al., 2024), the MERCK SAP likely prevented a complete, hormone-induced shutdown of development, thereby reducing the physiological burden and supporting successful germination.

In summary, MERCK seedlings executed a finely tuned adaptive program. Downregulation of *PER45* conserved auxin pools by limiting ROS-driven auxin catabolism, while induction of *STM* and *KNAT2* reinforced meristem competence to sustain organ formation under ionic and oxidative stress. Upregulation of *ABI1* restrained ABA overactivation, maintaining signaling activity without triggering growth arrest. Limiting *BGLU* activity further buffered against excessive ABA release, reducing hormonal noise and preventing premature stress-escape responses. Together, these adjustments demonstrate that MERCK achieves resilience not through maximal defense activation but through selective modulation of key regulators, thus enabling stress protection without compromising developmental continuity. The broader implication is that the chemical nature of the polymer governs the initiation adaptation versus a maladaptive transcriptional program in plants. By coordinating peroxidases, ABA regulators, and meristem maintenance genes in a targeted manner, MERCK uncouples stress responses from growth inhibition, exemplifying a general strategy in plant stress biology where resilience arises from integrated control of hormonal, developmental, and redox networks.

#### 4.2.2 B. natural-based SAPs

In contrast to the targeted response seen with fossil-based SAPs, the natural-based (ABG) triggered a gene expression signature characteristic of severe, uncontrolled stress. This included massive induction of genes in the ABA signaling pathway and the reprogramming of core developmental regulators. Such broad activation aligns with a state of high physiological stress and explains the observed impacts on seedling development. In this regard, we identified a set of 11 highly relevant DEGs (e.g., *MZB77, WRKZ33, CIPK17*, or *STz*) that were significantly downregulated in ABG. Many are linked to stress adaptation, suggesting that ABG not only activates canonical defense programs but also compromises key components required for maintaining developmental resilience.

Hormonal and developmental regulators were strongly affected. *MYB77* (*C07P45630.1*), a member of the MYB TF superfamily, integrates ABA–auxin signaling and promotes lateral root growth under stress (Zhao et al., 2014). Its downregulation in ABG seedlings is likely associated with high rates of abnormal germination, suggesting impaired auxin-mediated recovery. A second regulator, *UGT74E2* (*A08P36340.1*), an auxin-modifying enzyme responsive to hydrogen peroxide and essential for drought and salinity tolerance (Tognetti et al., 2010; Hopson et al., 2022; Rehman et al., 2018; Ma et al., 2024), was also downregulated. Together, reduced *MYB77* and *UGT74E2* expression likely destabilize auxin–ABA crosstalk. Furthermore, *NCED3* (*C05P52880.1*), a key enzyme in ABA biosynthesis (Muchlas et al., 2023; Wang et al., 2023), was significantly downregulated. This suggests that ionic stress in ABG interfered with a drought-induced ABA accumulation, reducing protective hormone pools.

Key transcriptional defense activators were also repressed. The *WRKY* family plays a central role in regulating plant stress (Khoso et al., 2022; Li et al., 2025b). In arabidopsis, *WRKY33* (*AT2G38470*) acts as a transcriptional hub for salt and hypoxia responses via salicylic acid (SA) and jasmonic acid (JA) mediated defense pathways (Zhang et al., 2022; Yang et al., 2025). Its rapeseed ortholog (*C03P25210.1, WRKY33*) normally enhances tolerance through MPK3-dependent phosphorylation and activation of downstream defense genes (Chen and Zhang, 2024; Yang et al., 2025). In ABG seedlings, however, *WRKY33* was downregulated, indicating impaired defense activation. Similarly, downregulation of RHL41 (A03P11470.1), a zinc finger TF required for drought and salinity tolerance (Malyukova et al., 2022; Samarina et al., 2020), was observed. Furthermore, *STZ* TFs (*C03P74520.1, A09P38370.1*), which balance growth–defense tradeoffs in root meristems (Yu et al., 2025) and enhance drought tolerance when overexpressed (Mukhopadhyay et al., 2004; Sakamoto et al., 2004; Dhar et al., 2024; Qin et al., 2024a), were strongly downregulated; this loss of transcriptional control likely impaired SA, JA, and ABA-dependent stress responses.

Stress signaling networks also showed inhibition. The calcium sensor *CIPK17* (*C08P53500.1*), commonly upregulated under salt stress to regulate ion homeostasis (Ding et al., 2022; Qin et al., 2024b), was significantly downregulated in ABG seedlings, suggesting disrupted Ca^2+^-dependent signaling. In addition, *PCaP1* orthologs (*A01P11960.1, C01P14230.1*), calcium-binding proteins critical for cytoskeletal remodeling, root hydrotropism, and microtubule depolymerization during stress (Lian et al., 2025; Giovannoni et al., 2021), were also downregulated. Their downregulation suggests that cytoskeletal dynamics and cell elongation were compromised, consistent with the observed developmental abnormalities.

Finally, the antioxidant defense system was compromised. Ascorbate peroxidase genes are enzymatic ROS scavengers (Kaur et al., 2021; Sun et al., 2023; Yang et al., 2022; Feki et al., 2024), yet the APX-related gene *A03P63220.1* remained weakly expressed in ABG seedlings despite evidence of elevated oxidative stress. This suggests inhibition of enzymatic detoxification and a compensatory reliance on non-enzymatic antioxidants, such as phenolic compounds.

Whereas ABG seedlings collapsed into a state of hyperactivated defense and impaired development, the treatment broadly downregulated multiple protective pathways, including hormonal regulation, transcriptional activation, calcium-mediated signaling, and antioxidant defenses. This coordinated inhibition might have deprived seedlings of critical adaptive mechanisms and produced a distorted stress response in which defense programs were overactivated while equally essential regulatory and developmental pathways were silenced. Such a molecular imbalance is in line with the high stress sensitivity, abnormal morphology, and germination failure observed under ABG. In particular, the loss of auxin-ABA crosstalk, impaired calcium signaling, and failure to induce antioxidant enzymes could restrict the plant’s ability to integrate environmental signals with developmental needs, leaving seedlings unable to re-establish homeostasis under stress.

## Data Availability

The datasets presented in this study can be found in online repositories. The names of the repository/repositories and accession number(s) can be found below: https://figshare.com/, https://doi.org/10.6084/m9.figshare.30165079.v1.
