# Supplementary figures and images for: Superabsorbent polymers seed coatings modulate transcriptomic and physiological responses to drought in rapeseed
Source: Front Plant Sci. 2026 Feb 11;17:1711479. doi: 10.3389/fpls.2026.1711479 (PMC12932477; doi:10.3389/fpls.2026.1711479)

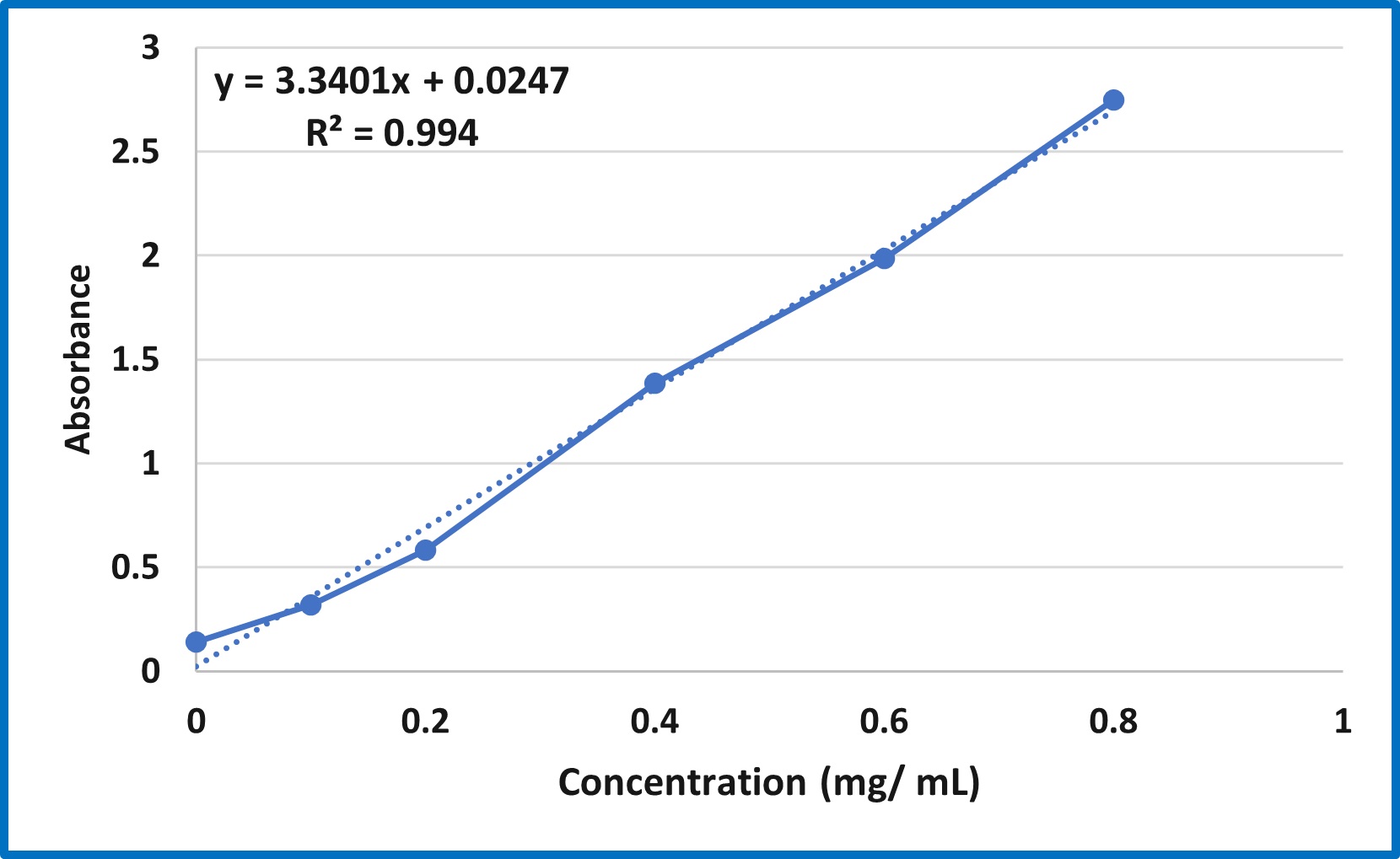

Supplement: Supplementary file 1 [file Image1.jpeg]
