# Supplementary material for: Superabsorbent polymers seed coatings modulate transcriptomic and physiological responses to drought in rapeseed
Source: Front Plant Sci. 2026 Feb 11;17:1711479. doi: 10.3389/fpls.2026.1711479 (PMC12932477; doi:10.3389/fpls.2026.1711479)
Supplement: Supplementary file 2 [file DataSheet1.pdf]

Supplementary Material

1 SUPPLEMENTARY DATA

1.1 TPC calibration curve

One gram of gallic acid was dissolved in 100 mL of 95% CH_3OH, resulting in a 1 % gallic acid solution (10 mg/mL) designated as the standard solution. To construct a standard gallic acid curve, dilutions (0.1, 0.2, 0.4, 0.6, 0.8, and 1 mg/mL) were prepared in methanol form the first standard solution (Solution 1). Each dilution (0.1 mL) was mixed successively with 0.5 mL of water and 0.1 mL of Folin-Ciocalteu reagent, followed by a 6-minute incubation period. Subsequently, 1 mL of 1 % NaOH and 0.5 mL of distilled water were introduced to the reaction mixture. The absorbance was recorded spectrometrically after 90 min at 760 nm Utilizing the standard gallic acid curve and regression equation, the total phenolic content of the extracts was calculated based on $y = 0.0352 + 3.2098\ x$ ($R^2 = 0.9976$), where y is the absorbance and x is the concentration of dilutions, as shown in Figure S1.

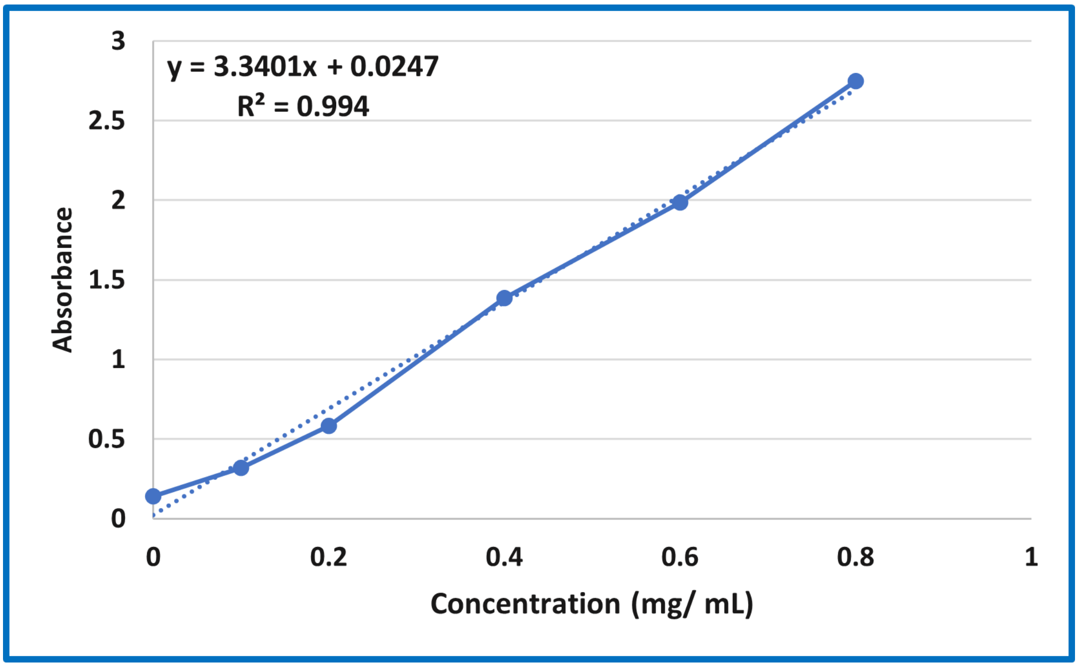

Figure S1. Standard curve of galic acid: Calibration curve to measure the TPC concentration in plant samples.

1.2 Quality Assessment of mRNA-Seq Data

To assess sequencing data quality, mRNA-seq read statistics were compiled for all rapeseed seedling samples under different SAP treatments (Table S1). For each sample, the total number of raw reads, reads retained after quality control, the percentage of reads passing filters, and alignment rates to the reference genome are reported. These metrics confirm the high quality and reliability of the sequencing data used for downstream transcriptomic analyses.

Table S1. mRNA-seq Read and Alignment Summary .Summary of mRNA-seq read statistics for rapeseed seedlings under different SAP treatments. Pre-filtering and post-filtering columns indicate total raw reads and reads retained after quality control, respectively. "Reads passed filters (%)" represents the proportion of reads retained after filtering, and "Alignment rate (%)" indicates the percentage of filtered reads successfully aligned to the reference genome.

!ht

Sample	Treatment	Pre-filtering	Post-filtering	Reads passed filters(%)	Alignment rate (%)
RABG901	ABG	122,787,040	121,926,014	99.30	96.6
RABG903	ABG	132,880,174	131,810,144	99.20	97.2
RCN602	CN	155,740,962	153,430,968	98.51	97.9
RCN603	CN	194,985,208	191,520,914	98.22	97.0
RCS701	CS	171,454,268	171,454,268	98.41	97.6
RCS702	CS	163,712,504	161,023,300	98.35	97.3
RSWT801	SWT	128,489,644	128,489,644	98.07	98.0
RSWT802	SWT	132,260,474	132,260,474	99.27	96.6
RMERCK1002	MERCK	124,524,054	123,336,362	99.04	97.9
RMERCK1003	MERCK	133,719,836	132,657,006	99.21	97.1

1.3 Common DEGs across all SAPs

The UpSet plot (Figure 3B) revealed twelve DEGs shared among all SAP treatments. As detailed in Table S2, these genes were significantly expressed in each SAP condition relative to CN. This conserved expression pattern suggests the presence of a core drought-responsive transcriptional program activated across SAP treatments, despite broader differences in their transcriptomic profiles.

Table S2. Common DEGs among all SAPS. The table lists common DEGs across all SAPs. The table lists DEGs shared across all treatments. Where available, Arabidopsis thaliana orthologs and their gene names are provided for functional context. Negative LFC values indicate downregulation (green), and positive values indicate upregulation (red) relative to CS. Asterisks (*) denote significant differences in LFC based on Benjamini–Hochberg–adjusted p-values (padj < 0.05) and $|\text{LFC}| > 1$

Gene ID	Arabidopsis ortholog	Gene Name	Log2FoldChange			
			CN vs. CS	MERCK vs. CS	ABG vs. CS	SWT vs. CS
A04P33570.1	AT2G44950	HUB1	-0.66	-2.32*	-1.04*	-1.56*
A05P40030.1	AT3G08770	LTP6	0.02	3.83*	3.31*	3.84*
A07P19270.1	AT2G28790		0.02	2.18*	1.68*	2.86*
A09P16230.1	AT1G62500	DEG27	0.03	5.66*	3.89*	4.67*
C01P08890.1	AT4G30170		0.00	-5.96*	4.24*	2.71*
C02P27790.1	AT1G70510	KNAT2	0.04	2.41*	2.91*	2.51*
C02P57670.1	AT5G28640	AN3	0.03	3.11*	2.10*	3.25*
C03P26110.1	AT2G39310	JAL22	0.02	4.69*	2.41*	3.44*
C03P68370.1	AT1G66760	DTX9	0.04	1.61*	1.92*	2.18*
C06P07920.1	AT1G52400	BGLU18	0.03	2.81*	3.84*	3.28*
C09P19670.1	AT1G62500	DEG27	0.03	4.53*	2.64*	3.34*
C09P19820.1	AT1G62360	STM	0.02	5.01*	4.28*	4.74*
